# South African speech-language therapists’ practices regarding feeding tube placement in people with advanced dementia

**DOI:** 10.4102/sajcd.v69i1.927

**Published:** 2022-12-09

**Authors:** Mariaan Cloete, Esedra Krüger, Jeannie van der Linde, Marien A. Graham, Sarveshvari B. Pillay

**Affiliations:** 1 Department of Speech-Language Pathology and Audiology, Faculty of Humanities, University of Pretoria, Pretoria, South Africa; 2 Department of Science, Mathematics and Technology Education, Faculty of Education, University of Pretoria, Pretoria, South Africa

**Keywords:** tube feeding, advanced dementia, speech-language therapists, dysphagia, beliefs, practices, South Africa, electronic survey

## Abstract

**Background:**

Studies related to tube feeding in people with dementia (PWD) remain a contested topic, neglecting the importance of speech-language therapists’ (SLTs) role in dysphagia management. Furthermore, SLT practices and beliefs regarding tube feeding in people with advanced dementia in an upper-middle-income country, such as South Africa, are unexplored.

**Objective:**

This study aimed to determine the practices and beliefs of SLTs in South Africa regarding tube feeding placement in PWD.

**Method:**

A self-compiled online survey was distributed using social media platforms and was completed by 83 South African SLTs with experience in swallowing and feeding management of PWD.

**Results:**

Most SLTs (78.8%) strongly believed they play a vital role in the decision-making regarding feeding tube insertion in PWD. This role is often met with several challenges, such as limited support from other healthcare professionals. Speech-language therapists with more experience and increased involvement in palliative care appeared to be more confident in supporting and counselling families of PWD on tube feeding. Many SLTs still recommend tube feeding despite its known negative consequences for PWD.

**Conclusion:**

The findings indicate a need for continued professional development for South African SLTs on feeding decisions in advanced dementia to increase knowledge and confidence in clinical practice. Speech-language therapists require guidelines by professional bodies and further dialogue amongst healthcare professionals to guide difficult feeding decisions in people with advanced dementia.

## Introduction

Approximately 86% of people with dementia (PWD) will experience swallowing and eating difficulties during the last 6 months of life (Gieniusz et al., 2018; Newman, Ray, Woodward, & Glass, 2020) because of progressive underlying neurodegeneration of the swallowing system (Rösler et al., 2015; Rumbach, Coombes, & Doeltgen, 2018). If left undiagnosed or untreated, oro-pharyngeal dysphagia (OPD) leads to aspiration pneumonia, a leading cause of death in PWD (Chen et al., 2018; Manabe et al., 2017; Rogus-Pulia et al., 2018).

Complex feeding decisions arise when advanced dementia is reached, and oral feeding is considered unsafe or does not meet nutritional needs (Chen et al., 2018; Payne & Morley, 2018; Schwartz, 2018). Advanced dementia is a state wherein a person’s mental and physical abilities deteriorate to a complete dependence on daily living tasks, such as personal care needs, including eating and swallowing, and severe constraints in verbal communication (Ijaopo & Ijaopo, 2019). Healthcare professionals often rely on speech-language therapists (SLTs) for their expertise in assessing and managing swallowing and feeding disorders to provide evidence-based judgement regarding enteral nutrition (Berkman, Ahronheim, & Vitale, 2019; Schofield, Dittborn, Huxtable, Brangan, & Selman, 2021). Speech-language therapists may adopt an alternative approach, recommending feeding tubes (FT) because of the risk of OPD and the perceived advantages of FT, namely improved nutrition and hydration, decreased risk of aspiration-pneumonia, sarcopenia and frailty in PWD (Berkman et al., 2019; Newman et al., 2020). Tube feeding is an alternative method for people who have difficulty swallowing and obtaining adequate oral intake to meet their body’s energy requirements (Ijaopo & Ijaopo, 2019).

There is a dearth of evidence regarding FT placement in PWD in South Africa. Clinical practice requires direction from international guidelines. The consensus amongst European professional organisations (European Society of Parenteral and Enteral Nutrition [ESPEN], 2016; National Institute of Health and Care Excellence [NICE], 2006) advises avoiding tube feeding in PWD (American Geriatrics Society Ethics, Clinical, & Models of Care, 2014; NICE, 2006). Tube feeding has been shown not to improve quality of life, nutritional status or comfort in end-of-life care (Faraday et al., 2021; Mori, Naito, Nakane & Tohara, 2019). Carers often rely on advance care plans (ACP) to guide decision-making for a person with dementia. The Health Professions Council of South Africa’s (HPCSA) palliative care ethical guideline document encourages patients to write down their treatment preferences in an advanced directive (AD), which is similar to a living will (HPCSA, 2019). This document serves as a written nomination of a surrogate in accordance with the *National Health Act*, which requires a person to make decisions on their behalf if they become incapacitated. However, AD or ACP remains controversial as the documentation could be revised. The implementation of ACP, particularly regarding swallowing and feeding, remains a topic of discussion in geriatric clinical practice (Schofield et al., 2021; Sellars et al., 2019).

There are also considerations of harm versus benefit. People with dementia who do not require end-of-life care may benefit from FT placement; this practice might seem futile in the case of those with advanced dementia (Chen et al., 2018). Some authors (Coutts, 2019; Newman et al., 2020) have highlighted that FT insertion does not harm the patient but may contribute to a higher mortality risk after insertion (Kenny & Singh, 2015). The main disagreement exists because of the mismatch between clinical practice and evidence-based guidelines, as withholding artificial nutrition and hydration can evoke a controversial response when candidacy for FT placement is questioned (Kochovska et al., 2020).

The decision to forego FT placement is preferably directed by an ACP (Berkman et al., 2019; Douglas et al., 2018). Forty per cent of PWD face decisions regarding intensive procedures, such as tube insertion (Kelly, Cumming, Kenny, Smith-Merry, & Bogaardt, 2018; Torke et al., 2020), and the need to make these decisions typically occurs when advanced dementia symptoms are severe, and decision-making capacity has deteriorated (Manabe et al., 2017). Less than 50% of newly diagnosed PWD initiate the ACP. Without ACPs, preferences of PWD are unknown, and family members are left overwhelmed with life-limiting decisions (Douglas et al., 2018). Consequently, families tend to rely on the assessment findings and clinical skills, such as SLTs, to decide on feeding measures for PWD (American Geriatrics Society Ethics et al., 2014; Berkman et al., 2019). Speech-language therapists’ beliefs and practices regarding feeding decisions for PWD warrant further investigation (Douglas et al., 2018).

Beliefs and practices about decisions regarding tube feeding are often morally and ethically driven by an intricate and implicit value system, including healthcare professionals’ age, gender, and culture, and their knowledge of evidence-based practice, ethnic background, and religious views (Chen et al., 2018; Douglas et al., 2018). External perspectives on the decision to commence with tube insertion are available, but little is known regarding SLTs’ practices and beliefs concerning the implementation of FT (Schwartz, 2018). There is a growing need to examine the state of practice, specifically in an upper-middle-income country with lower-income settings, such as South Africa (Kelly et al., 2018). Seventy-eight per cent of older adults requiring alternative approaches, such as tube feeding, live in lower-to-middle-income countries (LMIC). The absence of position statements may leave a gap for error in judgement (Connor, 2014; Vose, Kesneck, Sunday, Plowman, & Humbert, 2018).

Speech-language therapists in South Africa face limited resources and overwhelming caseloads (HPCSA, 2018). The HPCSA and the South African Speech, Language and Hearing Association of South Africa (SASLHA) do not currently have resources, such as policies or guidelines available for SLTs outlining the feeding practices for persons with advanced dementia. Pillay (2020) explored the current state of service provision in 2018 and found that 86% of SLTs worked in private practice whilst 14% worked in the public sector, emphasising the disparities between service delivery in different contexts. According to the HPCSA, the SLT to patient ratio is 1:18 000 (Coutts, 2019). This staggering population ratio mostly consists of vulnerable citizens presenting with concomitant and non-communicable diseases (Audain, Carr, Dikmen, Zotor, & Ellahi, 2017). South Africa faces a quadruple burden of disease exacerbated by poverty, high unemployment, socioeconomic disparity and an inefficient health system (Modjadji, 2021). Consequently, South African SLTs are increasingly faced with PWD with additional diseases, such as HIV and tuberculosis, which affect treatment and prognosis (Andrews & Pillay, 2017). As a result, the South African environment can be complicated and demanding for SLTs (Coutts, 2019).

Further investigation is warranted to gain insight into the factors influencing the decision-making of SLTs. This study aimed to determine the current beliefs and practices of SLTs in South Africa when considering FT placement in PWD. By probing SLTs’ perspectives, the study may provide insight and contribute to evidence-based practice for SLTs and other healthcare professionals regarding tube feeding in PWD in lower-to-middle-income settings (Druml et al., 2016).

## Methods

### Study design and setting

A prospective mixed-methods descriptive survey design was employed. An online social platform advertised the survey, accompanied by an electronic link. Electronic surveys may render valuable information seeing as it reaches a broad audience who may be dispersed across a large geographic area (Leedy & Ormrod, 2016).

### Process

A self-compiled electronic survey was compiled based on the published work of Shega et al. (2003), Sharp and Shega (2009), O’Reilly and Walshe (2015) and Vitale et al. (2011). The survey comprised 32 questions, of which 2 were mandatory and determined eligibility for participation in the questionnaire. The survey consisted of qualitative and quantitative data. Participants had to be English-proficient, qualified SLTs with current experience of, or have managed feeding and swallowing disorders in PWD in the last 5 years. Qualtrics™ XM software was used to distribute the self-compiled electronic survey to SLTs on social media platforms, and the survey was open for 1 month. Purposeful sampling was used. The questionnaire was distributed to respondents with a particular interest in PWD and dysphagia, known to currently serve or who have experience with the PWD population (Leedy & Ormrod, 2016). Purposive sampling allowed the researcher to select knowledgeable and reliable respondents, from the authors’ network base of SLTs, to provide information that would answer the research question. A sample of 83 respondents was recruited prospectively.

A pilot study was conducted by requesting two qualified experienced SLTs who fit the inclusion criteria, to evaluate the survey’s content and scrutinise the phrasing of the questions and the clarity of questions to prevent any ambiguity. Ambiguous questions might confuse respondents, resulting in misleading data (Leedy & Ormrod, 2016). Feedback was received from the SLTs regarding ease of completion, avoiding ambiguity and overpowering information provided during the survey, which might result in vague responses. The questionnaire was adjusted, adapted and amended to be sent out as a pilot study to an independent SLT who fit the inclusion criteria.

### Data analysis

Data were analysed using the Statistical Package for Social Sciences (SPSS) version 26.0 in collaboration with a statistician. Open-ended questions were coded thematically using qualitative content analysis (Vaismoradi et al., 2013). An exploratory factor analysis (EFA) was conducted, and Cronbach alpha values were calculated to determine the internal consistency of items in the questionnaire. The survey’s reliability was determined using Cronbach alpha values. The generally agreed-upon lower limit for Cronbach alpha is 0.70, although some researchers advocate that a value as low as 0.60 is acceptable in general (Daud, Khidzir, Ismail, & Abdullah, 2018; Zhan, Wei, & Hong, 2021) and in exploratory research (Hair, Black, Babin & Anderson, 2009). Firstly, an EFA was conducted, and four factors were identified. The Kaiser–Meyer–Olkin Measure of Sampling Adequacy (KMO) and Bartlett’s test of sphericity were performed to determine whether the data were suitable for factor analysis. The KMO (which should ideally be above 0.5) and Bartlett’s test of sphericity (of which the *p*-value should ideally be less than 0.05) showed evidence that dimension reduction could be achieved (KMO = 0.686, Bartlett’s test of sphericity *p* < 0.001). Each factor was named, and the Cronbach alpha value was computed for each factor (Table 1). The Cronbach alpha values indicate reliability for Factors 1–3 but not for Factor 4. Although the Cronbach alpha value of Factor 4 is below 0.6, it is well-known that the more items a factor has, the higher the Cronbach alpha. Authors (Briggs & Cheek, 1986; Pallant, 2001) have suggested that inter-item correlations be used to establish reliability when the factor has few items; Factor 4 has only two items. The recommendation is that an inter-item correlation should be between 0.1 and 0.5 because values lower than 0.1 indicate that a single total score could not adequately represent the complexity of the items, and higher than 0.5 means the items on the factor tend to be overly redundant. The inter-item correlation between the two items of Factor 4 equals 0.465 with a *p* < 0.001, indicating the correlation is statistically significant, establishing reliability for Factor 4. Furthermore, to establish construct validity, convergent validity (items belonging to the same factor should correlate highly) and discriminant validity (items not belonging to the same factor should have low correlations) should be established (Heale & Twycross, 2015). Although not all details are shown here for conciseness, convergent and discriminant validity have been established as items belonging to the same factors correlate stronger than items belonging to different factors.

**TABLE 1:** Factors and corresponding Cronbach alpha coefficients.

Factor	Number of items	Cronbach alpha
Factor 1: Area of practices	7	0.906
Factor 2: Frequency of involvement and the level of confidence	7	0.862
Factor 3: Tube feeding and quality of life	3	0.855
Factor 4: Counselling and support	2	0.534†

† , Although the Cronbach alpha coefficient for Factor 4 is unacceptable (< 0.6), since Factor 4 only contains two items, the Cronbach alpha coefficient is not considered the best statistical test to establish reliability of it, but, rather, an inter-item correlation coefficient should be used; this is discussed in more detail in the data analysis section.

The frequency distribution allowed further exploration of how different groups responded to the various factors. Differences between two independent groups were analysed using the Mann–Whitney (MW) test, and differences between three or more independent groups were tested using the Kruskal–Wallis test. *P*-values less than 0.05 indicated a statistical significance between these groups, and an *ad hoc* MW test was used following the Kruskal–Wallis test. For missing values, pairwise deletion was used over listwise deletion. The latter leads to smaller sample size and lower statistical power as the entire record is excluded from analysis if a single value is missing (Raaijmakers, 1999).

### Ethical considerations

The study obtained institutional ethical clearance from the Faculty of Humanities’ Research Ethics Committee at the University of Pretoria for the study protocol HUM004/1219 on 20 March 2020. Voluntary informed consent was obtained from respondents by providing a hyperlink of the informed consent document in the questionnaire.

## Results

One hundred and eighty-two potential respondents received an invite-over-email for the individuals who did not have social media access; only 42.2% (*n* = 35) agreed to participate. A total of 83 responses were recorded and statistically analysed. Respondents were 21–50 years of age, and of the 47 who answered the specific question regarding services to PWD, 85.1% (*n* = 40) currently provided services to PWD (Table 2). Interestingly, two participants, a doctor and a healthcare professional, also participated in the survey. However, these results were excluded based on the inclusion criteria. The largest number of respondents, 30.1% (*n* = 25), worked in private practice, followed closely by 28.9% (*n* = 24) respondents working in private hospitals providing in- and out-patient services to PWD, residential care facilities, 20.5% (*n* = 17), and 19.3% (*n* = 16) worked in rehabilitation facilities. Almost half of the respondents, 44.7% (*n* = 21), graduated 5 or less than 5 years ago.

**TABLE 2:** Respondents’ characteristics (*n* = 83).

Biographical questions where participants could only select one option	*n*	%
**Age (years) (*n* = 47)†**		
21–30	31	66.0
31–40	11	23.4
41–50	5	10.6
**Services rendered to PWD (*n* = 47)†**		
Yes	40	85.1
No	7	14.9
**Culture (*n* = 83)**		
Do you belong to a specific culture?		
Yes (did not elaborate)	8	9.6
Yes (elaborated)	21	25.3
No	8	9.6
I prefer not to disclose	46	55.4
**University (*n* = 47)†**		
University of Cape Town	8	17.0
University of Pretoria	23	48.9
University of the Witwatersrand	6	12.8
University of KwaZulu-Natal	4	8.5
Stellenbosch University	6	12.8
**Graduated (*n* = 47)†**		
< 5 years ago	21	44.7
6–10 years ago	17	36.2
11–15 years ago	3	6.4
> 16 years ago	6	12.6
**Qualification (*n* = 47)†**
Speech-Language Therapist (SLT)	31	66.0
Speech-Language Therapist and Audiologist (SLT/A)	14	29.8
Other	2	4.3
**Province where service is rendered (*n* = 43)†**		
Eastern Cape	2	4.7
Free State	3	7.0
Gauteng	24	55.8
KwaZulu-Natal	3	7.0
Mpumalanga	2	4.7
Northwest	1	2.3
Western Cape	8	18.6
**Clinical time per week (*n* = 46)†**		
0–2 h	17	37.0
3–5 h	18	39.1
6–8 h	2	4.3
> 8 h	9	19.6
**Biographical questions where participants could select more than one option** †		
**Current context involved‡ (*n* = 100)**		
Private practice	25	30.1
Private hospitals providing in-and out-patient services to PWD	24	28.9
Home for the aged/Residential care facility	17	20.5
Rehabilitation facilities	16	19.3
University-based clinics	5	6.0
Tertiary hospitals	5	6.0
Secondary level hospitals, that is, district hospitals	4	4.8
Primary level institutions	4	4.8

PWD, people with dementia.

† , Some instances of missing values;

‡ , Respondents could select more than one option, which could reflect a total of more than *n* = 83 and percentages adding up to more than 100%.

## The perceived role of the speech-language therapists in people with dementia

### Specific role in feeding tube placement

From the total of 83 respondents, 36.1% (*n* = 30) identified their current role in assessing and treating *PWD who might have dysphagia*. The largest proportion of SLTs (37/47, 78.7%) strongly agreed that they have a role in decision-making for PWD who might require tube feeding assistance. Most SLTs (46/47, 97.9%) agreed that their scope of practice includes swallowing and feeding management in PWD and felt that their regulatory body specified this role. Two areas in the multiple-answer question indicated the most pertinent roles of the SLT in treating PWD: *consulting with the individual, family and caregivers* involved in the care of the PWD 54.2% (*n* = 45) and 54.2% (*n* = 45) agreed to *assessing the individual’s swallowing and feeding ability*. From the multiple answer question, 49.4% (*n* = 41) of SLTs believe the SLTs’ primary role in their current professional setting was perceived as providing *diagnosis and assessment of feeding and swallowing abilities.*

### Beliefs and practices in the professional setting

In this section, the open-ended responses were analysed using thematic analysis from which themes emerged. The SLTs’ beliefs and practices were based on two identified themes that influenced FT placement in PWD: *best practice* for the PWD and the decision to implement tube feeding remains *patient dependent.* Most SLTs indicated they would recommend FT placement, but concerns were raised regarding the maintenance of tube feeding post-insertion. These concerns led to a feeling of uncertainty in palliative care practices, as seen by the following:

‘Unfortunately, palliative care practices are loosely defined in South Africa, and we do not have access to palliative care specialists at the hospital’. (Respondent 1, SLT, qualified within the past 5 years)

Table 3 outlines the roles of the speech-language therapists. Interestingly, more than half of the SLTs believed FT placement remains the most viable option for PWD and would recommend tube feeding to a family member. However, contrary to evidence-based practices (Ayman et al., 2017; Gieniusz et al., 2018), SLTs often succumb to external pressures and are often confronted or contested by three common aspects that often challenge FT placement decisions. These aspects are listed below, together with respondents’ corroborative statements:

1. Families’ insight, perception, and culture:
  ‘Families would do anything to keep a family member alive, even if tube feeding is not the optimal medical decision. The unrealistic expectation of families.’ (Respondent 2, SLT, qualified within the past 5 years)
2. Speech-language therapists’ bio-ethical concerns and belief system of the necessity for FT placement:
  ‘Ethical dilemmas when a poor prognosis is given. Upon discharge, the families often [*feel*] emotional and traumatised, with limited to no optimal care facilities upon discharge.’ (Respondent 3, SLT, experienced)
3. The lack of support from other healthcare professionals:
  ‘I think different allied health professionals have different opinions. The physicians only consider certain factors and not always look at the client holistically.’ (Respondent 2, SLT, qualified within the past 5 years)

**TABLE 3:** Roles of the speech-language therapists (*n* = 83).

Service delivery by SLTs to PWD	*n*	%
**Your regulatory body stipulates the role of feeding and swallowing (*n* = 47)†**		
Yes	46	97.9
No	1	2.1
**SLTs have a role in decision-making and care for the individuals who require assistance with feeding/may require tube feeding (*n* = 47)†**		
Strongly agree	37	76.7
Agree	3	6.4
Strongly disagree	7	14.9
**Challenges often experienced in decision-making regarding tube feeding (*n* = 57)** ‡		
Family members’ insight, perception and cultural values	22	38.6
SLT’s personal beliefs system and bio-ethical concerns	18	31.6
Lack of support from other healthcare professionals	17	29.8
**Perceived contribution of the SLT regarding tube placement in PWD**		
**Specific SLT roles identified in tube feeding placement for PWD (*n* = 69)** ‡		
Assessment and treatment of PWD who might have dysphagia	30	36.1
Education	16	19.3
Quality of life	10	12.0
Ensure safe oral intake	6	7.2
Collaborative teamwork	7	8.4
**SLT role when providing services to a PWD (*n* = 214)** ‡		
Consulting with the patient, family and caregivers involved in the care of the PWD	45	54.2
Communicating and liaising with other members of the healthcare team regarding progress and issues limiting or enhancing patient care in PWD	44	53.0
Planning of intervention goals with the PWD	40	48.2
Explain to the PWD that the goal is to improve and maintain comfort during mealtimes	40	48.2
**Considerations guiding decision-making (*n* = 101)** ‡		
Age and cognitive level of functioning	23	27.2
Severity and progression of the dementia	22	26.5
Quality of life of and level of support and/or post-tube insertion	20	24.1
Family’s wishes and cultural beliefs	18	21.7
Swallowing abilities	18	21.7
**SLTs’ contribution when working with PWD in your current clinical or professional setting (*n* = 152)** ‡		
Provide diagnosis and/or assessment of the PWD’s feeding and swallowing abilities	41	49.4
Provide referrals to other specialists when making recommendations for further intervention to the healthcare team	34	41.0
Provide a consultative role during family meetings discussing tube feeding insertion in PWD	32	38.6
Screen PWD for feeding and swallowing difficulties to provide intervention strategies	25	30.1
SLT should consult the PWD with the physician upon arrival to rule out feeding and swallowing problems	20	24.1

SLT, speech-language therapists; PWD, people with dementia.

† , Some instances of missing values;

‡ , Respondents could select more than one option, which could reflect a total of more than *n* = 83 and percentages adding up to more than 100%.

Table 4 summarises responses that were statistically significantly different (*p* < 0.05) per factor (identified in Table 1).

**TABLE 4:** Statistically significant differences between responses per factor (*n* = 83).

Descriptive statistic	Response										Mann-Whitney statistic	*p*
	Yes	No	Option selected	Option not selected	0–2 h	3–5 h	0–2 h	6 or more hours	21–30 years	31–40 years		
**Factor 1: Area of practices**												
Question 3: Do you currently render any feeding and swallowing services to PWD?											58.5	0.014
Mean	4.48	3.45	3.86	4.37	-	-	-	-	-	-	-	-
SD	0.732	1.351	0.617	0.927	-	-	-	-	-	-	-	-
Median	4.71	3.86	-	-	-	-	-	-	-	-	-	-
IQR	0.679	2.143	-	-	-	-	-	-	-	-	-	-
Question 13_3: In which context are you providing these services? Secondary level hospitals, that is district hospitals and regional hospitals											34.0	0.045
Mean	-	-	3.86	4.37	-	-	-	-	-	-	-	-
SD	-	-	0.617	0.927	-	-	-	-	-	-	-	-
Median	-	-	4.00	4.71	-	-	-	-	-	-	-	-
IQR	-	-	1.143	0.714	-	-	-	-	-	-	-	-
Question 13_5: In which context are you providing these services? Rehabilitation facilities											130.5	0.008
Mean	-	-	4.75	4.11	-	-	-	-	-	-	-	-
SD	-	-	0.311	1.039	-	-	-	-	-	-	-	-
Median	-	-	4.86	4.43	-	-	-	-	-	-	-	-
IQR	-	-	0.393	1.039	-	-	-	-	-	-	-	-
Question 13_7: In which context are you providing these services? Private hospitals providing in-and out-patient services to PWD											178	0.035
Mean	-	-	4.53	4.13	-	-	-	-	-	-	-	-
SD	-	-	0.853	0.940	-	-	-	-	-	-	-	-
Median	-	-	4.86	4.36	-	-	-	-	-	-	-	-
IQR	-	-	0.571	1.000	-	-	-	-	-	-	-	-
Question 15: Have you undertaken any further education in feeding and swallowing in PWD-related continuous professional development events?											171.0	0.028
Mean	4.64	3.93	-	-	-	-	-	-	-	-	-	-
SD	0.357	1.207	-	-	-	-	-	-	-	-	-	-
Median	4.79	4.29	-	-	-	-	-	-	-	-	-	-
IQR	0.571	1.429	-	-	-	-	-	-	-	-	-	-
**Factor 2: Frequency of involvement and level of confidence**												
Question 3: Do you currently render any feeding and swallowing services to PWD?											66.0	0.026
Mean	4.15	3.39	-	-	-	-	-	-	-	-	-	-
SD	0.618	0.950	-	-	-	-	-	-	-	-	-	-
Median	4.21	3.43	-	-	-	-	-	-	-	-	-	-
IQR	0.821	1.143	-	-	-	-	-	-	-	-	-	-
Question 14: How much clinical time do you spend providing feeding and swallowing intervention to PWD on a weekly basis?											90.0	0.037
Mean	-	-	-	-	3.63	4.18	-	-	-	-	-	-
SD	-	-	-	-	0.794	0.414	-	-	-	-	-	-
Median	-	-	-	-	3.57	4.14	-	-	-	-	-	-
IQR	-	-	-	-	0.929	0.607	-	-	-	-	-	-
-	-	-	-	-	-	-	-	-	-	-	37.0	0.008
Mean	-	-	-	-	-	-	3.63	4.43	-	-	-	-
SD	-	-	-	-	-	-	0.794	0.769	-	-	-	-
Median	-	-	-	-	-	-	3.57	4.57	-	-	-	-
IQR	-	-	-	-	-	-	0.929	0.714	-	-	-	-
Question 15: Have you undertaken any further education in feeding and swallowing in PWD-related continuous professional development events?												
Mean	4.23	3.80	-	-	-	-	-	-	-	-	-	-
SD	0.658	0.735	-	-	-	-	-	-	-	-	-	-
Median	4.29	4.00	-	-	-	-	-	-	-	-	178.0	0.041
IQR	1.036	0.857	-	-	-	-	-	-	-	-	-	-
**Factor 4: Counselling and support**												
Question 7: What is your current age?												
Mean	-	-	-	-	-	-	-	-	4.03	4.55	-	-
SD	-	-	-	-	-	-	-	-	0.657	0.650	-	-
Median	-	-	-	-	-	-	-	-	4.00	5.00	90.5	0.017
IQR	-	-	-	-	-	-	-	-	0.500	1.000	-	-

Note: Factor 3: Tube feeding and quality of life did not show any significant results; there were no statistically significant differences, and therefore Factor 3 is not represented in Table 4.

PWD, people with dementia.

Question 3 showed a statistically significant difference between whether an SLT currently renders any swallowing and feeding services to PWD and their score for Factor 1 (*area of practices* [*p* = 0.014]) and Factor 2 *(frequency of involvement and level of confidence*) [*p* = 0.026]), respectively. Respondents who provided feeding and swallowing services to PWD had statistically significantly higher scores for Factor 1 and Factor 2 than those who did not. Current clinical practice and frequency in treating swallowing and feeding challenges contribute to higher confidence in rendering services to PWD. Respondents aged 31–40 years agreed more strongly (*p* = 0.017) than those aged 21–30 years, with statements formed by Factor 4 (*counselling and support*). When investigating the context in which these services were delivered, respondents working in *private hospitals providing in- and out-patient services to PWD* (*p* = 0.035) and in *rehabilitation facilities* agreed significantly (*p* = 0.008) more strongly with statements forming Factor 1 (*area of practices*) than those respondents who did not render services at these hospitals and facilities. In contrast, the group of respondents from *secondary level hospitals, that is, district hospitals and regional hospitals,* tended to be significantly less in agreement with these statements forming Factor 1 (*p* = 0.045).

When investigating clinical time, respondents who indicated 6 or more hours per week (*p* = 0.008) and those who indicated 3–5 h per week (*p* = 0.037) agreed significantly more strongly with statements that formed Factor 2 (*frequency of involvement and the level of confidence*) than those who indicated 0–2 h of clinical time per week. Respondents who have undergone further education and training agreed more strongly with the statements forming Factor 1 (*area of practices* [*p* = 0.028]) and those forming Factor 2 (*frequency of involvement and level of confidence* [*p* = 0.041]) than respondents who have not had further training.

### Team members involved

The level of involvement from other multidisciplinary team (MDT) members and SLTs’ satisfaction with MDT participation when caring for PWD were examined (Table 5). Most respondents, 87.2% (*n* = 41), are part of an MDT. The multiple-answer question identified that the three most frequently nominated team members were the SLT, 54.2% (*n* = 45); dietician, 54.2% (*n* = 45); and physiotherapist, 51.8% (*n* = 43). Of those respondents who are part of MDT teams, less than half (20/42, 47.6%) are satisfied with their own level of involvement.

**TABLE 5:** Level of involvement of team members (*n* = 83).

Involvement in the multidisciplinary team	*n*	%
**Are you a part of the multidisciplinary team (MDT) involved in decision making for care and intervention for PWD? (*n* = 47)†**		
Yes	41	87.2
No	6	2.8
**Are you satisfied with your level of involvement in the healthcare team for PWD? (*n* = 26)†**		
Responded yes	20	76.9
Responded no	6	23.1
**Team members involved in your clinical or professional team (*n* = 318)** ‡		
Dietician	45	54.2
Speech-language therapist	45	54.2
Physiotherapist	43	51.8
Nurse	38	45.8
Physician	37	44.6
Occupational therapist	35	42.2
Social worker	29	34.9
Neurologist	27	32.5
Psychologist	19	22.9

MDT, multidisciplinary team; PWD, people with dementia.

† , Some instances of missing values;

‡ , Respondents could select more than one option, which could reflect a total of more than *n* = 83 and percentages adding up to more than 100%.

Open-ended questions revealed two related themes: *inconsistent involvement of other healthcare professionals,* and *limited inclusion in the decision-making of alternative feeding methods.* The identified themes highlighted the need for more collaboration between healthcare professionals treating PWD because most SLTs feel excluded when decisions regarding FT placement are taken, as demonstrated by the following statement:

‘Treating physicians do not always value our input and make their own decision, which is often uninformed. The extent to which I am involved depends on the referring doctor.’ (Respondent 4, SLT, experienced)

### Continuous professional development

Approximately 55.3% (*n* = 26) of respondents had invested in PWD-related continuous professional development activities (CPD) prior to this survey.

Qualitative information gathered from open-ended questions in Table 6 revealed the following themes: the need for increased *CPD opportunities or events, clear guidelines in the healthcare context* and *online training.* Interestingly, *evidence-based research* was the main focal point, followed by *information regarding ethical concerns relating to FT placement in PWD,* as seen in the following statement:

‘It is ethically an exceedingly difficult decision. I would appreciate more training and discussion regarding the right decision based on international guidelines and research.’ (Respondent 4, SLT, experienced)

**TABLE 6:** Further education (*n* = 83).

Continuous professional development	*n*	%
**Would you like to know more regarding the management of feeding and swallowing in PWD? (*n* = 47)†**		
Yes	42	89.4
No	5	10.6
**Themes identified: participants’ knowledge needs (*n* = 33)** ‡		
EBP research required to form part of or conduct research	19	22.9
More information regarding ethical concerns	12	14.5
Future analysis regarding tube feeding in PWD	2	2.4
**Themes identified: The preferred medium of CPD delivery identified by participants to accommodate knowledge needs (*n* = 148)** ‡		
CPD	35	42.2
Clear guidelines in the healthcare setting (protocol)	32	38.6
Online training	26	31.3
Interactive workshops	22	26.5
More information regarding ethical concerns	12	14.5
Face-to-face training	16	19.3
Practical supervision	5	6.0

CPD, continuous professional development; PWD, people with dementia.

† , Some instances of missing values;

‡ , Respondents could select more than one option, which could reflect a total of more than *n* = 83 and percentages adding up to more than 100%.

## Discussion

The study found that the perceived role of the SLT is to assess, diagnose and treat OPD in PWD, followed by providing appropriate ongoing and alternative feeding within SLTs’ specific area of expertise and scope of practice. Speech-language therapists tend to focus on person-centred care in deciding what PWD want to eat and drink, but with limited systematic support to direct decision-making. Similarly, whilst most SLTs mentioned that decision-making mechanisms would help with nuanced feeding decisions, official guidelines remain absent.

Speech-language therapists spending more clinical time (6+ h per week) rendering services to PWD appeared to have more confidence in FT placement decisions than those who indicated less clinical time (Andrews & Pillay, 2017; Schwarz, Coccetti, & Cardell, 2020). Speech-language therapists with more experience and a higher frequency of involvement in managing PWD appeared to be more confident in supporting and counselling families of PWD on FT. Speech-language therapists who are sporadically involved in decision-making practices regarding FT placement in PWD may submit to external influences, such as emotional responses from families (Coutts, 2019; Schwarz et al., 2020), or input from other healthcare professionals, because of their undefined role within the MDT (Hawksley, Ludlow, Buttimer, & Bloch, 2017; Kelly et al., 2018; Krikheli, Mathisen, & Carey 2018). Speech-language therapists rely less on their clinical expertise (Schwarz et al., 2020), and the tendency to succumb to pressure may be exacerbated by limited physical and human resources and a lack of clinical experience.

Speech-language therapists mentioned that the PWD’s *age and cognitive status* and the *severity and progression of dementia* play a role in FT placement. A further consideration is SLTs’ bio-ethical concerns and the legal implications of FT placement (Kelly et al., 2018). Speech-language therapists’ expertise helps them act as intercessors between the PWD, other healthcare professionals and family members to reach a consensus regarding FT placement for PWD (Chahda, Mathisen, & Carey, 2017). A lack of other healthcare professionals’ support in this decision-making is evident, further highlighting the additional pressure SLTs face in daily clinical practice. MDT involvement in FT decisions is often complicated by families’ insights, perceptions and cultural values (Berkman et al., 2019; McHutchison, Miles, Spriggs, & Jayathissa 2018). Family involvement is crucial as they remain the primary decision-makers but tend to favour tube feeding even when it is discouraged by healthcare teams (Rumbach et al., 2018). Because of the unique linguistic and cultural diversity in local settings, misunderstandings regarding care might compel patients’ families to consult alternative medical practitioners accepted according to cultural beliefs (Druml et al., 2016; Pascoe, Klop, Mdlalo, & Ndhambi, 2018; Salifu, Almack, & Caswell, 2021). In a multicultural and multilingual setting, such as South Africa, clear communication between families and healthcare providers is essential (Pascoe et al., 2018).

South African SLTs appear to rely more on clinical experience than research-based information (Andrews & Pillay, 2017). Speech-language therapists in this study believed that their roles are congruent with their professional healthcare settings’ expectations of the role of the SLT. The SLT’s decision to agree to FT placement would be accepted amongst healthcare professionals if clear guidelines were available to inform practice, which is the case for neither South Africa nor high-income countries (Andrews & Pillay, 2017; McHutchison et al., 2018). New Zealand and the United Kingdom are the only countries currently developing supportive documents to assist in making decisions regarding FT placement (Kelly et al., 2018; Schwarz et al., 2020). Inconsistency exists in SLTs’ clinical practice because of a mismatch between SLTs’ belief systems and the policies of the SLTs’ governing bodies (Berkman et al., 2019). There is currently no guideline provided by the South African Speech-Language and Hearing Association regarding FT placement decision making for SLTs in South Africa. The National Department of Health (2017) has a current policy that states there is no structure or formal mechanism in the public health sector for introducing a multidisciplinary team of health workers, healthcare professionals and social workers to implement better palliative care procedures. This context of the survey confirms the SLTs’ perceived limited interaction between multi-disciplinary team setups. However, HPCSA’s palliative care ethical guidelines (2019) state that only treatment options that are scientifically supported and reasonably expected to yield the intended benefits should be recommended and provided by healthcare practitioners providing palliative care.

A clinical void seems to exist, and SLTs may discard their clinical knowledge of the best practice (Schwarz et al., 2020) to accommodate PWD’s family members’ wishes (Schwarz et al., 2020) and other healthcare professionals’ opinions, resulting in sub-optimal decision making (Berkman et al., 2019; Vose et al., 2018). Although the HPCSA’s palliative care ethical guidelines (2019) advise healthcare professionals to consider the possible benefits versus the risks of palliative care in subsequent interventions, formal guidelines for SLTs may assist in decision making for FT insertion in the later stages of dementia or serve as a preventative guide in the earlier stages (Kochovska et al., 2020; Sellars et al., 2019). Further research is thus warranted.

Providing alternative feeding options to patients with advanced dementia is an ethical obligation, and such provision is a fundamental human right (Vose et al., 2018). South Africa has the highest inequality of health services amongst all LMICs, which further highlights the need for guidelines to ensure best practices concerning PWD (Andrews & Pillay, 2017; Pillay, Tiwari, Kathard, & Chikte, 2020). Graduate SLTs expressed a need for further evidence-based education consisting of events, workshops or online CPD activities. Continued research using focus groups may provide insight regarding FT decisions and address ethical issues in the advanced stage of dementia in a resource-constrained setting, such as South Africa (Coutts, 2019).

In this study, most SLTs indicated that they provided services in the private sector. Therefore, responses are more reflective of this sector and less representative of SLTs in the public healthcare sector. Regrettably, SLTs’ service delivery to individuals with advanced dementia and their families accessing public healthcare services could not be comprehensively described, and this topic warrants further exploration. One respondent’s response to the question exploring decision-making challenges regarding tube feeding was as follows:

‘In the public sector: no access to PEGs, catheter tubes are placed, poor amenities at home, for example, no access to clean running water, and education levels/ language barrier/ understanding of the care of PEG tubes.’ (Respondent 4, SLT, experienced)

Although most respondents graduated 5 or fewer years ago and could therefore be expected to have a broader perspective, they still believed that FT placement for PWD remains the most viable option and would recommend FT placement to a family member. The significant decrease in the number of survey participants as their years since qualification increased could indicate a number of factors, possibly being less active on social media platforms or fewer SLTs providing this specialised service to people with advanced dementia. Comfort feeding or pleasure feeding was not mentioned as an option to consider in the quality of life of PWD. Although these practices pose some challenges, they are patient-dependent and currently regarded by many as best practices for PWD (Schwarz et al., 2020). A patient-centred approach with individualised decision-making is ideal (Orlandoni et al., 2020).

Further research should investigate local SLTs’ implementation of comfort feeding with PWD (Andrews & Pillay, 2017; Kelly et al., 2018). Knowledge gained from this study could be used in undergraduate training and continued professional development of SLTs.

## Conclusion

The use of FT in PWD remains a matter of contention. Speech-language therapists need further research to drive contextually specific guideline development that may aid decision making in the case of PWD to ensure high-quality patient-centred care. The findings of this study may guide undergraduate training and continued professional development of South African SLTs. Future research endeavours may include conceptualising guidelines by professional bodies to aid healthcare professionals, such as SLTs in decision making about FT placement in PWD.

### Strengths and limitations

It may be that the SLTs who participated in this study most likely had a firm opinion, interest or belief in this topic. Recall bias might have occurred and influenced responses because of personal experience with a PWD. To the researchers’ knowledge, this survey was the first performed in a country encompassing both upper-middle-income and lower-income settings, and therefore the findings are valuable. Awareness about FT placement in PWD was raised, and SLTs may have recognised the need for further professional development regarding this matter. A substantial sample yielded valuable responses from SLTs from various South African settings, but missing data limit the findings.
